# Primary Aldosteronism Associated with Multiple Adrenocortical Micronodules in a Patient with Renal Cell Carcinoma

**DOI:** 10.1155/2020/2808101

**Published:** 2020-02-24

**Authors:** Kazuhito Oba, Yuko Chiba, Yoko Matsuda, Takeshi Kumakawa, Rie Aoyama, Miho Akahoshi, Seiji Hashimoto, Aya Tachibana, Koichi Toyoshima, Remi Kodera, Kenji Toyoshima, Yoshiaki Tamura, Takashi Nagata, Yuto Yamazaki, Hironobu Sasano, Atsushi Araki

**Affiliations:** ^1^Departments of Diabetes, Metabolism, and Endocrinology, Tokyo Metropolitan Geriatric Hospital Tokyo, Tokyo, Japan; ^2^Department of Pathology, Tokyo Metropolitan Geriatric Hospital, Tokyo, Japan; ^3^Department of Cardiology, Tokyo Metropolitan Geriatric Hospital and Institute of Gerontology, Tokyo, Japan; ^4^Department of Urology, Tokyo Metropolitan Geriatric Hospital, Tokyo, Japan; ^5^Department of Pathology, Tohoku University Graduate School of Medicine, Sendai, Japan

## Abstract

A 47-year-old woman with a history of diabetes mellitus (DM) and obesity was admitted to our hospital for glucose control. She was detected to have hypertension (HT) and diagnosed with primary aldosteronism (PA) based on the high level of aldosterone to renin ratio and the results of the upright furosemide-loading test according to the criteria of the Japanese Society of Hypertension (JSH) guidelines. Computed tomography revealed left renal tumor and adrenocortical adenoma. She underwent left nephrectomy and adrenalectomy. The pathological findings were clear-cell renal cell carcinoma (RCC) and nonfunctional adrenocortical adenoma. Her nonneoplastic adrenal tissue histologically revealed CYP11B2-positive multiple adrenocortical micronodules (MNs) and concomitant paradoxical hyperplasia of the zona glomerulosa. Therefore, MNs were thought to be responsible for PA in this patient. After surgery, HT was improved, and the result of upright furosemide-loading test after 12 months of surgery did not fulfill the criteria of PA according to the JSH guidelines. However, the adrenocorticotrophic hormone stimulation test was positive; considering the possibility of slight aldosterone overproduction from the right adrenal gland, the administration of spironolactone was started. Herein, we report a rare case of RCC in conjunction with PA histologically associated with MNs.

## 1. Introduction

Primary aldosteronism (PA) is a disorder characterized by the excessive production of aldosterone from the adrenal adenoma or hyperplasia, leading to sodium retention, potassium loss, and high blood pressure, thereby causing secondary hypertension, diabetes mellitus (DM), and cardiovascular disease [1]. Previous studies have shown that PA could also be complicated with various malignancies, in particular, renal cell carcinoma (RCC), the incidence of which is associated with aldosterone levels [2]. The aldosterone-mediated upregulation of K-RAS is considered to result in the activation of the Akt and Raf pathways, which leads to tumor proliferation [3], or oxidative stress and DNA damage caused by aldosterone [4] might promote the development of RCC; however, the mechanistic details have remained largely unknown.

In contrast, some previous studies revealed that RCC was associated with adrenal tumors, including unilateral or bilateral adrenal adenoma, adrenal hyperplasia, or metastasis [5–9]. Two cases of unilateral adrenal hyperplasia with contralateral RCC have been reported [10].

PA due to multiple adrenocortical micronodules (MNs) was first reported in 2002 [11]. Subsequently, monoclonal antibodies against cytochrome P450 11B2 (CYP11B2), which recognized the enzymes involved in aldosterone synthesis, were developed [12]. CYP11B2 immunohistochemistry is useful for definitive histopathological diagnosis to detect precisely the lesions responsible for PA, such as aldosterone-producing adenoma (APA), diffuse hyperplasia (DH), and MNs [13]. MNs are defined as the presence of CYP11B2-positive cortical micronodules in the CYP11B2-negative zona glomerulosa (ZG), which has been considered to develop from the aldosterone-producing cell cluster (APCC) [14].

Herein, we report a rare case of RCC in conjunction with PA exhibiting pathological findings of MNs.

## 2. Case Presentation

A 47-year-old woman with a history of DM and obesity (BMI, 41.7 kg/m^2^) was admitted to our hospital for the treatment of DM. Although she did not have a history of hypertension, her systolic and diastolic blood pressures were 144−169 and 82−98 mmHg, respectively, and she had a high aldosterone to renin ratio (ARR), as revealed by 0.9 ng/mL/h of plasma renin activity (PRA) and 201 pg/mL of plasma aldosterone concentration (PAC). Serum potassium and creatinine level were 3.9 mEq/L and 0.60 mg/dl, respectively. She underwent an upright furosemide-loading test; her PRA level after loading was 0.8 ng/mL/h and fulfilled the diagnostic criteria for PA according to the Japanese Society of Hypertension (JSH) guidelines [15]. Furthermore, her adrenocorticotropic hormone (ACTH) stimulation test (intravenous injection of 250 *µ*g of tetracosactide acetate) result was positive because of high PAC max/cortisol ratio (17.6; PAC and cortisol levels were 403 pg/mL and 22.9 *µ*g/dL, respectively) [16]. The ACTH-cortisol system was intact because the plasma ACTH level was 15.3 pg/mL; fasting and late-night serum cortisol levels were 10.7 *µ*g/dL and 3.7 *µ*g/dL, respectively; and the urinary cortisol level was 41.2 *µ*g/day. Serum dehydroepiandrosterone sulfate level was 140 ng/mL, which was within the normal range. Hence, she was diagnosed with hypertension due to PA.

**Figure 1:**
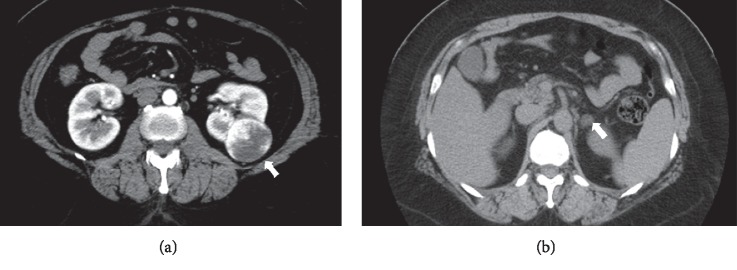
The computed tomographic (CT) findings of the left adrenal tumor. (a) Renal tumor in the arterial phase. Most of the left renal tumor was enhanced using contrast medium, except the necrotic area. (b) Adrenal tumor. The left adrenal tumor was a low-density mass having a diameter of 10 mm and was suspected as adrenocortical adenoma.

Abdominal computed tomography (CT) revealed a low-density tumor of 45 mm in diameter in the left kidney. In the contrast-enhanced CT, most part of the left renal tumor was enhanced in the arterial phase (Figure 1(a)) and washed out in the venous phase; furthermore, the tumor included a necrotic part, which was not enhanced by the contrast agent. This strongly suggested that the tumor was a RCC. Furthermore, a low-density left adrenocortical tumor of 10 mm in diameter was detected (Figure 1(b)). Adrenal scintigraphy showed no uptake in both the adrenal glands. The ACTH-stimulated adrenal venous sampling was unsuccessful and incomplete. The localization of PA was not known. After detailed explanation was provided, she decided to undergo surgery for the removal of both the left renal tumor and left adrenal gland.

**Figure 2:**
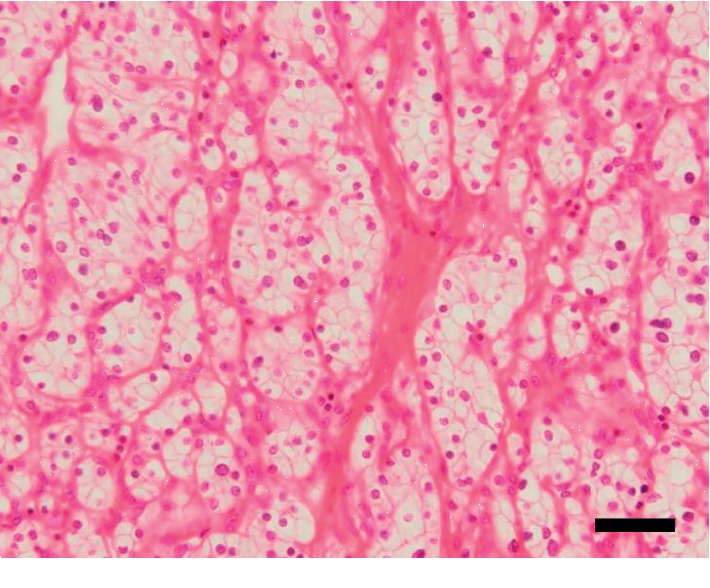
Histopathological findings of the renal cell carcinoma. Hematoxylin and eosin staining. The tumor cells had small round nuclei and clear cytoplasm with cell atypia. The tumor cells showed trabecular pattern with abundant vessels in the tumor stroma. These findings were consistent with grade 2 clear-cell renal cell carcinoma. Bar, 50 *µ*m.

**Figure 3:**
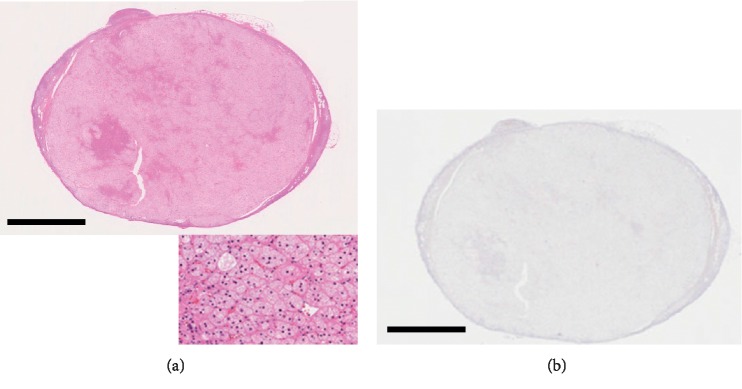
Histopathological findings of the adrenocortical adenoma. (a) Hematoxylin and eosin staining. The adrenocortical tumor was predominantly composed of clear cells. These characteristics are consistent with those of adrenocortical adenoma. Bar, 3 mm. (b) Cytochrome P450 11B2 (CYP11B2) immunostaining. The tumor cells were negative for CYP11B2. Bar, 3 mm.

She underwent left radical nephrectomy and left adrenalectomy. The resected left kidney included a well-circumscribed, solid, and yellow-to-red tumor measuring 43 × 39 × 29 mm. The renal tumor was well circumscribed with the fibrous membrane and was entirely composed of clear cells with relatively small round nuclei (Fuhrmann grade: Grade 2). The tumor cells were arranged in a trabecular or solid alveolar pattern with abundant vessels in their stroma (Figure 2). The renal tumor was histopathologically diagnosed as stage I, grade 2 clear-cell RCC (T1bN0M0). Macroscopically, the left adrenal tumor had xanthochromic appearance and measured 11 mm in its greatest dimension. Light microscopic examination revealed that the tumor was predominantly composed of clear cells (Figure 3(a)). Immunoreactivity of steroidogenic factor-1 was detected at the nuclear side of the tumor cells, suggesting that this tumor had originated from the adrenal cortex (data not shown) [17]. Based on the criteria of Weiss [18, 19], we histologically diagnosed the tumor as adrenocortical adenoma. This adenoma was negative for CYP11B2 immunoreactivity (Figure 3(b)), indicating that it did not have the ability to produce aldosterone. Therefore, we concluded that this adenoma was not responsible for PA.

**Figure 4:**
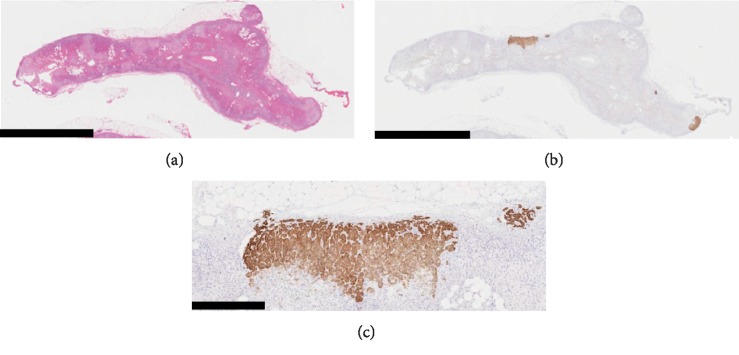
Histopathological findings of multiple micronodules and hyperplastic lesions in the left adrenal gland. (a) Hematoxylin and eosin staining. Multiple hyperplastic lesions in the left adrenal gland. Bar, 5 mm. (b) CYP11B2 immunostaining. Multiple CYP11B2-positive micronodules were detected predominantly at the subcapsular area of the adrenal cortex and showed preserved zonation. Many CYP11B2-negative hyperplastic cells were detected in the zona glomerulosa; paradoxical hyperplasia of the zona glomerulosa was noted. Bar, 5 mm. (c) CYP11B2 immunostaining. The magnified image of the CYP11B2-positive micronodules shown in (b). The CYP11B2 immunoreactivity waned from the subcapsular area to inward in the micronodules, in which zonation was preserved. Bar, 600 *µ*m.

In contrast, CYP11B2-positive MNs measuring less than 2 mm in their greatest dimension were detected predominantly at the subcapsular area of the adrenal cortex (Figure 4(a) and 4(b). The CYP11B2 immunoreactivity was predominantly noted at the subcapsular part of the micronodules and diminished inward. Based on the CYP11B2 immunoreactivity pattern in the micronodules, we considered that their zonation was preserved (Figure 4(c)); therefore, we diagnosed them as nonneoplastic adrenocortical lesions. The nonnodular part of the ZG morphologically revealed hyperplasia, but was negative for CYP11B2 immunoreactivity (Figure 4(a) and 4(b)), consistent with the paradoxical hyperplasia of the ZG, which is frequently detected in the adjacent adrenal cortex of APA [20]. Therefore, MNs were considered to be responsible for PA in this case.

Her postoperative course was uneventful, and she was discharged at 12 days after the surgery. During the follow-up of 6 months, her systolic and diastolic blood pressures and glucose control (HbA1c) were improved to 122−141 mmHg, 70−72 mmHg, and 6.0–6.5%, respectively. Her body weight had also reduced from 106.8 kg to 95.0 kg. However, after the operation, PRA level was still low (mean 0.7 ng/ml/h, range: 0.5−0.9 ng/mL/h), and PAC was relatively high (mean: 267 pg/mL, range: 182−351 pg/mL), so that ARR had a high level (mean: 452 pg/mL/ng/mL/h, range: 202−702 pg/mL/ng/mL/h) in the 2nd month after surgery. Then, spironolactone was administered and continued for 8 months.

She underwent an upright furosemide-loading test and an ACTH stimulation test 12 months after left adrenalectomy and 2 months after discontinuation of spironolactone. Her PRA level after loading was elevated to 3.1 ng/mL/h from 0.3 ng/mL/h (before loading) and did not fulfill the diagnostic criteria for PA as per the JSH guidelines [15]. However, the ACTH stimulation test was positive because of high PAC max/cortisol ratio (10.7; PAC and cortisol levels were 256 pg/mL and 24.0 *µ*g/dL, respectively). Considering the possibility of slight aldosterone overproduction from the right adrenal gland, the administration of spironolactone was started again.

## 3. Discussion

We experienced a rare case of RCC in conjunction with PA exhibiting pathological findings of MNs. PA patients are at an increased risk of developing RCC [2]. Lang et al. showed that 5 of 335 patients with PA had RCC (13% of all malignancies), whereas no RCC was detected in the hypertensive controls [2]. Patients with PA were reported to have higher oxidative stress, which could be reduced by treating PA [4]. Previous in vitro and animal model studies suggest that high aldosterone levels can cause oxidative stress, leading to DNA damage [21–23]. In our case, high aldosterone levels could have been involved in the development of RCC. Alternatively, unknown growth factors produced by RCC might promote the growth of adrenal tumors because RCC often produces hormones, growth factors, and inflammatory cytokines such as IL-6 [24].

Because PA is associated with cardio- and cerebrovascular complications and mortality [1], making accurate diagnosis and localizing PA are important because surgically correctable lesions are characterized as unilateral. Primary lesions in PA have been classified as APA, idiopathic hyperaldosteronism (IHA), unilateral adrenal hyperplasia (UAH), primary adrenal hyperplasia (PAH), adrenal cancer, and glucocorticoid-remediable aldosteronism [25]. In addition to these, Omura et al. reported 4 cases of PA due to unilateral MNs in 2002. They concluded that the clinical and pathologic characteristics of unilateral MNs are distinct from those of APA, IHA, UAH, and PAH. Furthermore, PA induced by MNs might be frequently overlooked because MNs cannot always be detected using the standard adrenal CT or scintigraphy [11]. In our case, we could not localize the lesion responsible for PA by using CT and scintigraphy. Because specific monoclonal antibodies, which can accurately distinguish the isoforms of CYP11B1 and CYP11B2 from each other, have been developed, aldosterone-producing cells can be identified using CYP11B2 immunohistochemistry [12], which is considered to be indispensable for the histopathological diagnosis of PA subtypes classified into APA, DH, or MNs [13].

APCC was first revealed as a CYP11B2-positive cell cluster within the ZG area in the nonpathological adrenal gland [26, 27]. However, APCC has been shown to induce PA by using CYP11B2 immunostaining [13]. The APCC from PA was found to be different from that in the nonpathological adrenal gland. The prevalence of somatic mutations in the aldosterone-driver gene in multiple APCCs that caused PA was considerably higher than that in the APCC in the nonpathological adrenal gland. Multiple APCCs might cause PA when 1% of the adrenal cortex becomes positive for CYP11B2 [14]. PA with unilateral or bilateral MNs has been extensively reported [14, 28–30]. Some MN cases have common genetic mutations in *CACNA1D*, *ATP1A1*, *ATP2B3*, and *KCNJ5* to those found in APA cases [14, 26]. MNs might be involved in the genesis of APA through these mutations. Since we did not conduct gene mutation analysis in our case, further research is necessary to clarify this point.

In this study, we did not conduct pathological investigation of the right adrenal gland. After the resection of the left adrenal gland, high blood pressure, hyperglycemia, and body weight control were improved. However, ARR and PAC levels remained high in contrast to low PRA levels after left adrenalectomy. Furthermore, the result of the ACTH stimulation test showed that the PA was not completely cured by left adrenalectomy although the result of upright furosemide-loading test was negative. The ACTH stimulation test has been reported to be useful for the diagnosis of PA with the sensitivity of 98% and the specificity of 91% [16]. These results suggest that the possibility of some overproduction of aldosterone from MNs, micro-APA, or DH from the right adrenal gland cannot be neglected.

Therefore, we started medical treatment with spironolactone after surgery to prevent cardiovascular events and renal function deterioration in the long term. Several studies indicated that both surgical and medical treatment of PA could prevent organ damage and decrease the risk of cardiovascular events and renal disease progression [31]. Careful follow-up is necessary to check for the recurrence of adrenal tumors as well as RCC.

In conclusion, we report a rare case of RCC in conjunction with PA exhibiting pathological findings of MNs. This case was considered to be valuable from the viewpoint of the pathogenesis and treatment of PA due to MNs.
